# Supplementary material for: Rights based approaches to sexual and reproductive health in low and middle-income countries: A systematic review
Source: PLoS One. 2021 Apr 29;16(4):e0250976. doi: 10.1371/journal.pone.0250976 (PMC8084237; doi:10.1371/journal.pone.0250976)
Supplement: S1 Table — (DOCX) [file pone.0250976.s001.docx]

**S1 Table:** **Risk of bias in cluster randomised controlled trials**

| Study | Randomisation process | Timing/ identification and recruitment of participants | Deviations from intended interventions | Missing outcome data | Measurement of the outcome | Selection of the reported result | Overall Risk of bias |
| --- | --- | --- | --- | --- | --- | --- | --- |
| Naved 2018 | Some concerns | High risk | Low risk | Low risk | High risk | High risk | High risk |
| Björkman 2009 | Some concerns | High risk | Some concerns | Some concerns | Low risk | High risk | High risk |
| Pandey 2007 | Low risk | High risk | Low risk | Some concerns | Low risk | Some concerns | High risk |
| Basu 2004 | High risk | High risk | Some concerns | High risk | High risk | Some concerns | High risk |

Risk of bias for cluster randomised trials assessed using the RoB-2 tool for cluster-randomised studies.[18]
