# Supplementary material for: Rights based approaches to sexual and reproductive health in low and middle-income countries: A systematic review
Source: PLoS One. 2021 Apr 29;16(4):e0250976. doi: 10.1371/journal.pone.0250976 (PMC8084237; doi:10.1371/journal.pone.0250976)
Supplement: S2 Table — (DOCX) [file pone.0250976.s002.docx]

**S2 Table: Risk of bias in non-randomised interventional studies**

| Study | Risk of bias due to confounding | Risk of bias in selection of participants into the study | Risk of bias in classification of interventions | Risk of bias due to deviations from intended interventions | Risk of bias due to missing data | Risk of bias in measurement of outcomes | Risk of bias in selection of the reported result | Overall risk of bias |
| --- | --- | --- | --- | --- | --- | --- | --- | --- |
| Guha 2012 | Moderate | Serious | Moderate | Low | No information | Serious | Serious | Serious |
| Rana 2012 | Moderate | Serious | Low | Low | Low | Serious | Moderate | Serious |
| Deering 2011 | Moderate | Serious | Moderate | Low | No information | Serious | Serious | Serious |
| Erasquin 2012 | Moderate | Serious | Moderate | Low | Low | Serious | Serious | Serious |
| Gangopadhyay 2010 | Moderate | Serious | Moderate | Low | Serious | Serious | Serious | Serious |
| Swendeman 2009 | Moderate | Serious | Low | Low | Serious | Serious | Serious | Serious |
| Halli 2006 | Moderate | Serious | Moderate | Low | Moderate | Serious | Serious | Serious |

Risk of bias for non-randomised intervention studies assessed using ROBINS-I tool for non-randomised studies.[19]
