# Supplementary material for: Rights based approaches to sexual and reproductive health in low and middle-income countries: A systematic review
Source: PLoS One. 2021 Apr 29;16(4):e0250976. doi: 10.1371/journal.pone.0250976 (PMC8084237; doi:10.1371/journal.pone.0250976)
Supplement: S3 Table — (DOCX) [file pone.0250976.s003.docx]

**S3 Table: Risk of bias in uncontrolled before-and-after studies**

Uncontrolled before-and-after studies based on two cross-sectional surveys were given an overall assessment of serious or critical risk of bias based on the authors’ judgement.

| Study | Serious or Critical risk of bias | Reason |
| --- | --- | --- |
| Metwally 2019 | Critical | No results adjusted for confounders. Different numbers of women in text and tables. No statistical test of significance. Different women before and after due to cross-sectional nature of study design but no age/demographic info so not possible to compare other characteristics. Surveys non-blinded. |
| Ratcliffe 2016 | Serious | Simple design. Limited confounders due to short timeframe. Response bias possible as response may be influenced by thinking about what they should write down due to setting. Retention of acquired knowledge may not last beyond that day. |
| Urada 2016 | Critical | Before/after on same day. High risk of response bias given that participants just had intervention (for instance intention to use condoms). Intention to use condoms is a proxy for condom use but may not predict condom use. Other questions on knowledge may be less prone to response bias. |
| Abuya 2015 | Critical | Overlapped with another evaluation of a voucher programme for reproductive health; not the same women in intervention and control; significant differences in education, health, and previous history of abuse or rape in baseline and end-line - i.e. multiple confounders. |
| Gurnani 2011 | Critical | No numbers given, no statistical test, before and after without any clarity whether those included actually received intervention. |
| Beattie 2010 | Critical | Multiple analyses of before/after, self-reported exposure, baseline undertaken after intervention started. |
| Ramesh 2010 | Critical | Surveys were anonymised so no way to tell if any of the same population in pre and post - therefore a large amount of confounding may be present, and any change may be due to differences in the populations that were surveyed. Not clear whether some participants may have been exposed to other interventions/whether they all did get exposed. |
| Reza-Paul 2008 | Critical | Similar to Ramesh et al, surveys anonymised so may or may not include some of the same participants before and after. Recall bias in terms of exposure to interventions. |
| Sinha 2008 | Serious/Critical | Multiple outcome measures, intervention at village level - multiple potential confounders. Sample selection was different for baseline and end-line, baseline and end-line survey questions differed, not adjusted for potential confounders. |
| Benzaken 2003/2007 | Critical | Methodology unclear, no adjustment of confounders, no indication intervention necessarily applied to population who were sampled, different year of follow up in results table and text (Table 4 and Table 5), unclear how population sampled. |
| Jana 1995/1998 | Critical | Little information given on methods - unclear how surveyed follow up, how participants were selected, no confounding factors measured/adjusted for, no demographics given. |
