# Supplementary material for: Rights based approaches to sexual and reproductive health in low and middle-income countries: A systematic review
Source: PLoS One. 2021 Apr 29;16(4):e0250976. doi: 10.1371/journal.pone.0250976 (PMC8084237; doi:10.1371/journal.pone.0250976)
Supplement: S2 File — (DOCX) [file pone.0250976.s005.docx]

**Search strategy used for MEDLINE:**

| # | Searches | # of results |
| --- | --- | --- |
| 1 | maternal health.tw. | 4526 |
| 2 | sexual health.tw. | 7125 |
| 3 | exp sexual health/ | 606 |
| 4 | sexually transmitted disease.tw. | 5710 |
| 5 | exp sexually transmitted disease/ | 329763 |
| 6 | sexually transmitted infection*.tw. | 10541 |
| 7 | exp Human immunodeficiency virus/ | 95639 |
| 8 | human immunodeficiency virus.tw. | 77091 |
| 9 | hiv.tw. | 268800 |
| 10 | exp gender based violence/ | 128 |
| 11 | gender based violence.tw. | 518 |
| 12 | exp Partner Violence/ | 8896 |
| 13 | domestic violence.tw. | 4927 |
| 14 | female genital mutilation.tw. | 866 |
| 15 | fgm.tw. | 754 |
| 16 | female circumcision.tw. | 382 |
| 17 | maternal mortality.tw. | 8334 |
| 18 | exp unplanned pregnancy/ or exp pregnancy outcome/ or exp unwanted pregnancy/ or exp pregnancy/ or Pregnancy Complications/ or Delivery, Obstetric/ | 870175 |
| 19 | pregnancy.tw. | 328265 |
| 20 | miscarriage.tw. | 8691 |
| 21 | contraception.tw. | 24338 |
| 22 | exp contraception/ | 26108 |
| 23 | condom.tw. | 13343 |
| 24 | healthcare attendance.tw. | 15 |
| 25 | healthcare access.tw. | 836 |
| 26 | adherence.tw. | 91013 |
| 27 | mental wellbeing.tw. | 401 |
| 28 | mental health.tw. | 106525 |
| 29 | Depression, Postpartum/ | 5112 |
| 30 | neonatal outcome*.tw. | 7697 |
| 31 | conviction.tw. | 2627 |
| 32 | legal action.tw. | 550 |
| 33 | right* based.tw. | 669 |
| 34 | right*-based.tw. | 669 |
| 35 | legal right*.tw. | 784 |
| 36 | civil right*.tw. | 964 |
| 37 | human right*.tw. | 7936 |
| 38 | exp human rights/ | 138382 |
| 39 | basic right*.tw. | 248 |
| 40 | patient right*.tw. | 703 |
| 41 | reproductive right*.tw. | 737 |
| 42 | wom* right*.tw. | 1035 |
| 43 | personal autonomy.tw. | 419 |
| 44 | quantitative.tw. | 491909 |
| 45 | analy*.tw. | 4658503 |
| 46 | intervention.tw. | 461967 |
| 47 | evaluat*.tw. | 2828632 |
| 48 | assess.tw. | 780818 |
| 49 | impact.tw. | 721882 |
| 50 | implement.tw. | 49050 |
| 51 | 1 or 2 or 3 or 4 or 5 or 6 or 7 or 9 or 10 or 11 or 12 or 13 or 14 or 15 or 16 or 17 or 18 or 19 or 20 or 21 or 22 or 23 or 24 or 25 or 27 or 28 or 29 or 30 | 1438535 |
| 52 | 31 or 32 or 33 or 34 or 35 or 36 or 37 or 38 or 39 or 40 or 41 or 42 or 43 | 146597 |
| 53 | 44 or 45 or 46 or 47 or 48 or 49 or 50 | 7649056 |
| 54 | 51 and 52 and 53 | 5550 |
