# Supplementary material for: Rights based approaches to sexual and reproductive health in low and middle-income countries: A systematic review
Source: PLoS One. 2021 Apr 29;16(4):e0250976. doi: 10.1371/journal.pone.0250976 (PMC8084237; doi:10.1371/journal.pone.0250976)
Supplement: Attachment — Submitted filename: Response to Reviewers.docx [file pone.0250976.s006.docx]

Dear Editorial Team,

Re: **Manuscript ID PONE-D-20-29838** – “Rights based approaches to sexual and reproductive health in low and middle-income countries: a systematic review”

We thank the editorial team and reviewers for taking the time to read our manuscript and for your helpful comments. We feel that by addressing your suggested changes, the paper is much improved, and we hope that you will agree.

Please find below a table outlining our response to each issue raised. Please note, the page numbers refer to the document ‘Revised Manuscript with Track Changes’.

| Comment | Reply |
| --- | --- |
| Editorial | |
| Please ensure that your manuscript meets PLOS ONE's style requirements, including those for file naming. The PLOS ONE style templates can be found at  <https://journals.plos.org/plosone/s/file?id=wjVg/PLOSOne_formatting_sample_main_body.pdf> and  <https://journals.plos.org/plosone/s/file?id=ba62/PLOSOne_formatting_sample_title_authors_affiliations.pdf> | The manuscript has been updated to ensure it fulfils PLOS ONE’s style requirements. |
| Please attach a Supplemental file of the results of the individual components of the quality assessment, not just the overall score, for each study included. Thank you. | Tables 3 and 4 (now S1 and S2 Tables) include the individual components of the quality assessment. Table 5 (now S3 Table) shows uncontrolled before-and-after studies for which we gave a serious or critical risk of bias, and we provide the rationale for each decision in Table 5. |
| Reviewer one | |
| The reviewed paper focused on rights-based approaches (from a rights holder perspective) to sexual and reproductive health (SRH) of adolescent girls and women in low and middle-income countries (LMICs). The authors found that right-based approaches seemed to be effective in the context of SHR. However, the examined approaches lacked evidence.  In total, I would suggest this paper to be published. The researched topic is of relevance to Global and Public Health. Further, the paper is well written and the different sections are coherent between each other. However, I would suggest minor changes, which will be stated in the following paragraphs, stating the chapters of the paper. | Thank you, we agree that this topic is of relevance to Global and Public Health and respond to your suggested changes as outlined below. |
| The abstract was written in a logical order and reflects the relevant aspects of the article. Adjustments: 1.) I would suggest stating the concrete age for the population, rather than refereeing to just words. That would be a more precise way to reflect the study population included in this study. | We have updated the abstract to state the population includes only females aged over 15 which we agree makes it more precise. |
| The introduction states the relevance of the topic rather well and is coherent in that the authors describe all relevant aspects regarding the current situation of sexual and reproductive health in LMICs and right-based approaches. Further, the authors create a connection between the two topics by describing the current state of the art. Hence, they validate why this review is of importance. Adjustments: none | Thank you for these helpful comments. |
|  |  |
| The methods are written with clear language and in a logical order. Adjustments:.  1.) I would recommend that the objectives be made more precise. The added value of the review should be made clear. | Thank you for this – we include both in the final paragraph of the introduction (pages 5-6), so have not included either in the methods. |
| 2.) While the appendix contains the PRISMA flow diagram and checklist, it was not indicated in the Methods section that the authors followed the PRISMA statement. I would suggest stating this at the beginning of the chapter. | This has been added to the methods (page 7). |
| 3.) By naming the search terms in the appendix, it is transparent how the authors obtained their results. Nevertheless, I would recommend, to briefly mention which subject areas are included in the search algorithm in the methods. | We have now added the subject areas of the search terms to the methods to clarify this (page 7). |
| 4.) Further, the authors describe the inclusion criteria in detail; however, do not this in a similar way for the exclusion criteria. To be more precise, I would empathize doing this. | We have added a section for “Exclusion criteria” in the methods (Page 9) to clarify this. |
| 5.) The inclusion criteria, also, include a bullet point as exclusion criteria, which is a little bit confusing to me | This has been moved to the “Exclusion criteria” section described above. |
| 6.) As already mentioned in the abstract section, I would suggest stating the age of the study population specifically. | The age of the population has been added to the inclusion criteria on Page 7. |
| The results are clearly arranged and correspond to the tables presented. The tables support the text and are comprehensible. Overall, the results support the conclusion of the paper. | Thank you. |
| Adjustments: 1.) Table 1. should be reviewed to coherent writing between the columns. For instance, in the column ‘population/setting (…)’ Metwalley et al. and Benzaken et al., the setting is stated directly (e.g. Egypt) and Naved et al. start with the heading (e.g. the setting). | Table 1 has been updated to ensure coherent writing between columns by including “Setting” and “Population” headings for each study, and numbering the intervention components and outcomes in the respective columns. |
| 2.) For table 3. and 4., it would be helpful to have the tool used for categorizing the risk of bias, as additional information underneath. That would help the reader to remember the underlying basis on which the categorization was made. | Thank you – this has been added to these tables (which can now be found as supplementary tables rather than in the text). |
| The discussion summarizes the most relevant results once again and puts them into a different context. Further, the authors describe the limits of their research. Adjustments: I have one question to ask. Why did the authors not use their definition of right-based approaches, to include a broader range of studies? I think that this question should be answered as well. | There is more than one definition of a rights-based approach, and rather than choose between these, we decided to choose the author’s definition. For the purpose of this systematic review, this made it more systematic and feasible for example, it allowed focussed searches (using words relating to “rights”) and allowed us to have an objective criteria for eligibility of interventions, rather than making subjective judgements about whether interventions fitted a broader definition or not.  We have recognised this in paragraph 2 of the limitations section. |
| The conclusion of this paper is, as already said, justified by the results. Further, it is short and precise. Hence, no adjustments are necessary. | Thank you for this. |
| Reviewer 2 | |
| Introduction  More explanation of the decision to exclude duty-bearers type approaches in this review is warranted. | For reasons of feasibility, we focused our systematic review on women in order to have a manageable number of papers and meaningful synthesis. This explanation has been added to the introduction (page 5). |
| The authors should present a definition for sexual and reproductive health. This will better highlight the gap in the literature that they are claiming the review fills. For example, does the authors’ definition of sexual and reproductive health include safe access to abortion? | We have now included a definition for sexual and reproductive health in the introduction (page 4). This definition does include the freedom to decide if, when, and how to reproduce and therefore safe access to abortion is included. |
| Methods  The methods states that there were no language restrictions. If that was the case, how many languages were include and who conducted the review of published studies in languages other than English? | One paper was identified in Mandarin and screened out by a University of Warwick colleague, Dr Yen-Fu Chen, following discussion with OO and MM – this has now been added to page 7 of the methods.  One included paper was in Portuguese (Benzaken et al. 2003) and was reviewed by the authors. We have now clarified this in the results (page 11). |
| The authors should describe their criteria for “rights-based approaches” further. Some of the included studies seem to be educational interventions so some distinction should be provided for when educational interventions are included vs. excluded. | This has been clarified in the methods (Page 8) to say that educational interventions were included if described by study authors as “rights-based” or related to “rights”. |
| It seems abortion was left off the acceptable outcomes list. One of the central arguments at the intersection of rights and sexual and reproductive health is women’s right to safe and equitable abortion care. Thus, this review is incomplete with regards to sexual and reproductive health and it is an oversight to leave out the abortion literature in its entirety. | We apologise that we did not explicitly list abortion in the list of primary outcomes. If we had identified a paper that included abortion as one of its outcomes we would have included it as we implicitly understand abortion to be part of sexual and reproductive health so we do not think that we have missed any relevant papers. We have added abortion to the discussion (page 32) to clarify that we did not identify abortion as an outcome in any included study. |
| Other outcomes to consider are impacts on OBGYNs and advanced practice providers who provide SRH care specifically. | As mentioned above, we chose to focus on women and girls in order to have a manageable number of papers and meaningful synthesis. |
| Results  Table 2 is not an effective way to present this information. A table that is similar to a “sample description” of all the included papers would be more effective and could include the distribution of studies across each of the outcomes, the study design, etc. | Based on previous publications (for example <https://pubmed.ncbi.nlm.nih.gov/30674487/>) we think this is an effective way to summarise the outcomes from the studies, which are further described in the text. |
| There should be an additional results table that aggregates information across the studies in a manner that is easy for readers to understand. If one is to quickly glance at the tables, they should be able to garner most of the information about this set of studies. Perhaps a table that further aggregates the information on types of “rights-based approaches” would be suitable. | Table 1 does aggregate the information in the studies. This is further repeated in the text.  We feel that with the changes to Table 1 suggested by reviewer 1, readers will be able to garner study information more quickly. |
| Tables 3 – 5 should be moved to the Appendices. The paragraph of results about the bias assessment can remain in the main text of the paper. Otherwise, the bulk of the tables are related to bias. | Tables 3-5 have now been moved to the Appendices (S1-S3 Tables). |
| Discussion  There are several grammatical errors in the discussion section and it should be revised with attention to detail. | Many thanks for highlighting this. We have updated the manuscript accordingly. |
| The fact that there is a similar review that focuses on maternity care is concerning, particularly, since 3 of the same studies are found in both reviews and there are sexual and reproductive health outcomes that are left out of this review. | This systematic review has expanded what existed already and provides a more complete picture of sexual and reproductive health. If you are referring to the omission of abortion from the explicit outcomes list, be reassured that we would have included any paper which included abortion as an outcome. |
| Another point of concern is that only 7 studies focus on non-FSW populations. Should this review be limited then to actor-based rights-based approaches to FSW SRH? It is not convincing to make any conclusions about the health of other groups based on seven studies, particularly, given the amount of bias reported in the studies as a whole. | We agree the fact that only 7 studies focus on non-FSW population is a concern. This is why we argue that “evidence is needed across a diverse range of populations (not limited to FSWs and maternity settings)” in our conclusion. One of the merits in this systematic review is exactly this: that we have identified a lack of evidence. |
| The approach undertaken in this review cannot be easily replicable. This is because most of the studies came from non-systematic approaches. But a large part of systematic reviews is the ability to reproduce the findings. | Reference screening is part of a systematic approach and would be replicable following our protocol. |
| Most of the discussion focuses on the limitations of this study and approach. Thus, it remains unclear what this review contributes to the existing literature. The limitations seem to overwhelm any merit that this work could have. The evidence to support the existence of such a systematic review is simply not there. | This systematic review synthesises evidence that suggests a rights-based approach may be promising. Any policy-maker or public health official planning an approach would be able to use our systematic review to justify taking this approach, while noting the need for further evidence to confirm the promise identified. We also show that there is a lack of evidence for these interventions in certain populations, and poor quality evidence for other populations. By synthesising the evidence base to illuminate the evidence gaps and what should be done in future research to improve the quality of the evidence base, this publication may move the field forwards. We have now clarified this in the conclusion (page 34). |
