# Supplementary material for: Rights based approaches to sexual and reproductive health in low and middle-income countries: A systematic review
Source: PLoS One. 2021 Apr 29;16(4):e0250976. doi: 10.1371/journal.pone.0250976 (PMC8084237; doi:10.1371/journal.pone.0250976)
Supplement: Attachment — Submitted filename: Response to Reviewers.docx [file pone.0250976.s007.docx]

Dear Editorial Team,

Re: **Manuscript ID PONE-D-20-29838** – “Rights based approaches to sexual and reproductive health in low and middle-income countries: a systematic review”

We thank the third reviewer, Dr Karen Hardee, for taking the time to read our manuscript and for your helpful comments. We feel that by addressing your suggested changes, the paper is much improved, and we hope that you will agree.

Please find below a table outlining our response to each issue raised. Please note, the page numbers refer to the document ‘Revised Manuscript with Track Changes’.

| Comment | Reply |
| --- | --- |
| Reviewer three | |
| The premise of this article is good – what is the evidence related to taking a human rights-based approach to SRHR. The article does not actually do this though, since a “human rights-based approach” seems to anything the authors of the studies included in the review said it was, including if the author said the intervention related to rights. I appreciate that the authors of the review said they were concerned that having their own definition might exclude some studies, but at the same time, the absence of a common definition of a rights-based approach (or what it means to be “related to rights), means that the reader doesn’t learn much about taking a rights-based approach to SRH – and how that differs from just labeling an intervention part of SRHR. If they simply said their intervention addressed rights, how can the authors of this review say that was that sufficient to include as a rights-based approach? | We have now added an example to support what we mean by “related to rights” (line 160, p8).  The intervention column of Table 1 describes what each paper included in terms of their intervention so that readers can interpret how well this aligns with a rights-based approach. We have added an explanation of whether authors referred to rights or described the intervention as a rights-based approach to this column for clarification.  Because there is more than one definition of a rights-based approach, rather than choose between these, we decided to choose the author’s definition. For the purpose of this systematic review, this made it more systematic and feasible. For example, it allowed focussed searches (using words relating to “rights”) and allowed us to have an objective criteria for eligibility of interventions, rather than making subjective judgements about whether interventions fitted a broader definition or not.  We have recognised this in paragraph 2 of the limitations section (lines 449-458, p31). |
| People have rights that they can claim, and rights standards and principles can be incorporated into programming. For example, individuals and couples have the right to decide freely and responsibly the number and spacing of their children, and they have the right to information and services to act on that right, with equity and free of discrimination and coercion. For contraceptive information and services, the rights principles and standards that need to be incorporated into programs are: availability, accessibility, acceptability and quality (AAAQ of the right to the highest attainable standard of health), informed decision-making, privacy and confidentiality, non-discrimination, participation and accountability (WHO, 2014, Ensuring Human Rights in Contraceptive Information and Services). The authors should describe what about “rights” and “rights-based approach” is noted in the studies included in the review. This would be an important contribution to the literature – what does it mean to take a rights-based approach to programming – in various areas of health. | We have detailed what rights-based language was used in the study reports (Table 1). Without contact with the study authors, it is not possible to elaborate as there is insufficient detail in the manuscripts to describe this further. We have described these challenges in the Discussion section (lines 409-415, page 29).  A further study could include contact with authors to elaborate what it means to take a rights-based approach to programming. |
| The authors note the SDG – Goal 3, but they equate it with SRHR, whereas Goal 3 is not ensuring that everyone can access SRHR – it is “Ensure healthy lives and promote well–being for all at all ages.” Targets under Goal 3 include reducing maternal mortality; ending preventable child deaths; ending or reducing AIDS other diseases; universal health coverage, affordable essential medicines, sexual and reproductive health care; vaccine research, and access to medicines. SRHR is measured in the SDG through two indicators – “Proportion of women of reproductive age (aged 15–49 years) who have their need for family planning satisfied with modern methods,” and the adolescent birth rate. https://sdg-tracker.org/good-health. So, according to the SDG, maternal health, HIV and GBV, which are the services covered in the review by the authors, are not technically part of SRH. That doesn’t negate the review – but the authors should not use the shorthand of “SRHR” to cover the topics they have included in the review – or they should say that they started with a broad list of SRH outcomes but only found relevant studies associated with a few of the SRH outcomes. | Thank you for this. We have removed the reference to Goal 3 and instead clarified our interpretation of Goal 5 (lines 15-17, page 2 and lines 66-68, page 4).  As explained in the discussion (lines 425-439, page 30), not all sexual and reproductive health outcomes that we were interested in were included.  As mentioned below, we have now clarified which SRH outcomes were included in lines 233-236, page 12. |
| The first sentence of the abstract: “By 2030, the Sustainable Development Goals aim to achieve human rights for all” is not accurate statement – the SDG are grounded in human rights, but the goals are not stated as “achieving human rights for all.” Suggest using the term “grounded in” – instead. <https://www.ohchr.org/Documents/Issues/MDGs/Post2015/SDG_HR_Table.pdf> | Thank you for this suggestion. We have updated the introduction of the abstract to reflect that the Sustainable Development Goals are grounded in human rights rather than aiming to achieve human rights for all (line 15, page 2). |
| The results part of the abstract notes that: rights-based interventions were effective for most included outcomes, but evidence was of poor quality. My reading of the table with results is that the findings were mixed. | Thank you for this – we have reworded to say that rights-based interventions were effective for some included outcomes (line 45, page 3). |
| Also, the limitations section of the paper indicates that it was often not possible to ascertain if the rights part of the intervention made the difference to outcomes – to me the bigger message from this review is that we don’t have common/consistent ways of incorporating rights into programming, and studies to assess the effect of taking a rights-based approach (or to address rights) are of low quality and more, better designed studies, with clear theories of change, are urgently needed. The authors make this point in the conclusion. | We have clarified in the limitations that there was no consistent approach to incorporating rights into programmes and agree that better designed studies are urgently needed. (Line 443-444, page 30-31) |
| The final sentence of the abstract: “Considerable risk of bias in all studies means results must be interpreted with caution. High-quality controlled studies are needed urgently in this area.” What is meant by “bias” in this sentence? | Risk of bias (i.e. systematic error) in this context is the risk that studies will overestimate or underestimate the true effect of the intervention based on an assessment of the quality of the study (for example, how the study selected participants, how the intervention was allocated, measures used to report outcomes etc). We assessed the risk of bias of included studies using the Cochrane Collaboration recommended tools (RoB 2 and ROBINS-I). |
| Introduction, lines 68-72. The distinction between rights vs. needs is likely to go over most readers heads – is it necessary? If so, it should be explained. | Thank you for highlighting this. We feel that this section is not necessary so have removed it. |
| Page 4, lines 76-79. The authors imply that UNFPA’s take on a human rights-based approach only has two components – those related to duty bearers and rights-holders. The reference (https://www.unfpa.org/human-rights-based-approach) includes a lot more than those 2 components. I suggest a more robust discussion of what a rights-based approach is, including reference to the UN Common Understanding of HRBA. | We have expanded our discussion of what a rights-based approach is (lines 85-92, page 5) including referencing the UN Common Understanding of HRBA. |
| Page 5, lines 83-88 focus on empowering women to claim their rights. That is important, but again, is not all there is to a human rights-based approach. What needs to be in place for women to claim their rights? | This has been expanded (lines 104-107, pages 5-6) |
| Page 11, Lines 203-204. Given the list of outcomes searched (shown on lines 122-123 - contraception, pregnancy, STIs, awareness of rights, violence and mental health), it would be good here to list the outcomes included in the 24 studies – this will help the reader understand why only some outcomes are included in the results section of the paper. | Thank you for this suggestion. We have now included the outcomes we included on lines 233-236, page 12. |
| It is not surprising that half of the studies are related to HIV – human rights were more actively and explicitly promoted as part of HIV programming than in other areas covered in the review. | We agree |
| Table 1 – I urge the authors to group studies rather than just listing them in chronological order. What about all of the HIV studies together, all of the maternal health studies together, etc. That will help readers look for patterns among the studies addressing each outcome. | Thank you for the suggestion – we have grouped by maternity, violence and HIV/STIs among sex workers |
| The title of Table 2 is misleading – the review did not assess the “effectiveness of a rights-based approach” – it included studies in which rights or rights-based were mentioned. As the authors say in the paper, there is no way of knowing what effect the “rights” or “rights-based” part had in the outcome. | We agree – we have changed the title of Table 2 to “Outcomes identified in included studies” |
| Page 23 – discussion of the outcomes. The authors note that of the studies measuring condom use as an outcome, “all of these studies were at serious, high or critical risk of bias.” Please explain in the methods section what is meant by “bias.” What is on pages 25-26, lines 322-327, should be moved into the methodology – and bias better explained that simply referring to supplemental tables (it is good to keep the supplemental tables, but readers need to better understand what bias is and how it was assessed in this review. | We have moved the suggested section to the methods, and clarified our interpretation of bias (lines 218-220, page 10). Our methods of risk of bias assessment are already discussed in lines 211-215 (we used publicly available tools recommended by the Cochrane Collaboration: The RoB-2 tool and ROBINS-I). |
